# Poly(caffeic acid) Redox Couple Decorated on Electrochemically Reduced Graphene Oxide for Electrocatalytic Sensing Free Chlorine in Drinking Water

**DOI:** 10.3390/nano13010151

**Published:** 2022-12-28

**Authors:** Srinivasan Kesavan, Deivasigamani Ranjith Kumar, Ganesh Dhakal, Woo Kyoung Kim, Yong Rok Lee, Jae-Jin Shim

**Affiliations:** School of Chemical Engineering, Yeungnam University, Gyeongsan 38541, Gyeongbuk, Republic of Korea

**Keywords:** caffeic acid, poly(caffeic acid), electrocatalytic reaction, free chlorine, tap water, drinking water

## Abstract

Regular water quality measurements are essential to the public water supply. Moreover, selective free chlorine (disinfectant) level monitoring without an interfering agent is necessary. The present work aimed to fabricate poly(caffeic acid) (p-CFA) coated on an electrochemically reduced graphene oxide (ERGO) surface for the selective detection of free chlorine. Electron microscopy and various spectroscopic techniques confirmed the p-CFA@ERGO/glassy carbon (GC) electrode. The p-CFA@ERGO/GC coated probe surface coverage was calculated to be 4.75 × 10^−11^ mol cm^−2^. The p-CFA@ERGO/GC showed superior catechol/*o*-quinone oxidation/reduction peaks for electrocatalytic free chlorine determination. The performance of the developed sensor electrode was outstanding, with an extensive range of free chlorine detection (20 μM to 20 mM), high sensitivity (0.0361 µA µM^−1^), and low detection limit (0.03 µM). The p-CFA@ERGO/GC capability of the realist water samples, such as the tested commercial and tap water, yielded a good range of recovery (from 98.5% to 99.9%). These values align with the standard *N,N*′-diethyl-*p*-phenylenediamine reagent method results.

## 1. Introduction

The quality of drinking water is a primary concern due to the many variables influencing human health. Chlorine is generally applied as a sanitizing agent in water process industries to regulate pathogenic microbes, particularly *Cryptosporidium* and *Escherichia coli*. Bleaching powder contains chlorine and is an effective oxidizing species for deodorizing and preparing different organic reagents [1]. The variable oxidation states of Cl_2_ compounds are used to sterilize polluted surroundings and drinking water [2]. Typically, sodium hypochlorite (NaOCl) is used for water purification in wastewater, swimming pool water, and drinking water sanitizer and purification [3]. The NaOCl and chlorine (Cl_2_) hydrolysis reaction mechanisms are presented below.

NaOCl → Na^+^ + OCl^−^ (1)

Cl_2_ + H_2_O → HOCl + HCl (2)

HOCl ⇌ OCl^−^ + H^+^ (3)

Hypochlorous acid (HOCl) dissociates to OCl^−^ (hypochlorite anion) and H^+^ (proton) at a pKa of 7.53. These OCl^−^ and HOCl species are called free chlorine [4]. Hypochlorous acid ionization depends mainly on the pH of the medium. In water with pH < 7.5, HOCl species exist in the majority, and HOCl/OCl^−^ is found over a pH range of 6–9. This variation is essential for water sterilization because HOCl is a stronger sanitizer than OCl^−^ [4]. HOCl/OCl^−^ is used as an oxidizing reagent for versatile organic functional group transformation and microorganism eradication in water [5,6]. 

According to the World Health Organization’s advice, the maximum limit of free chlorine allowed concentration in drinking water is 5 mg L^−1^ [7]. Beyond this concentration, various health disorders are possible, such as cancer, cardiovascular diseases, atherogenesis, neurodegenerative, and rheumatoid arthritis diseases [8]. In addition, it is a potential cause of anemia and central nervous system problems [9]. Hence, it is necessary to systematically monitor the free chlorine concentration in the water supply chain to the consumer or public municipal water source. Currently, various methods are used for free chlorine detections, such as potentiometry [10], chromatography [11], spectrophotometry [12], colorimetric methods [13], and chemiluminescence [14], respectively. In contrast, the electroanalytical approach has attracted increasing attention as a simple procedure that does not require a skilled person. In addition, it provides a rapid response, good sensitivity, and excellent selectivity. Recent reports of the boron-doped diamond electrode [15], graphite electrode [16], Au microelectrode [17], polymelamine on screen-printed carbon substrate [18], polydopamine-graphene oxide modified electrode [19], poly-Mn porphyrin-nano gold film-electrode [20], Prussian blue film-surfactant-assisted electrode [21], and Si chips covered with Au thin film-coated electrode [22] have been applied for the electrochemical detection of HOCl/OCl^−^ in water samples. These modified electrodes have limitations, such as OCl^−^ and oxygen reduction potential overlapped upon the electrochemical process, expensive electrode materials, high operating potential, and narrow concentration range detection. 

**Scheme 1:**
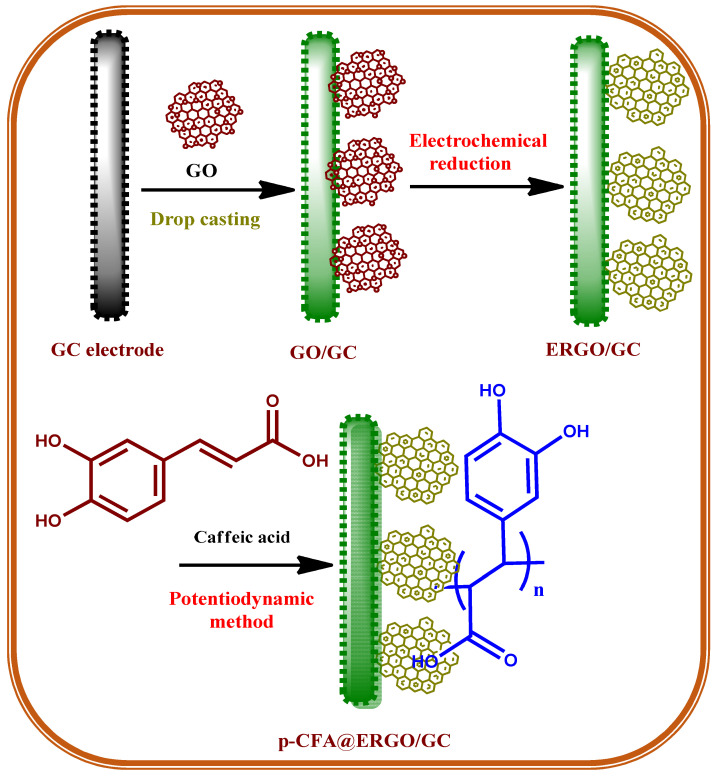
Schematic diagram of p-CFA@ERGO/GC free chlorine sensor electrode assembly.

**Scheme 2:**
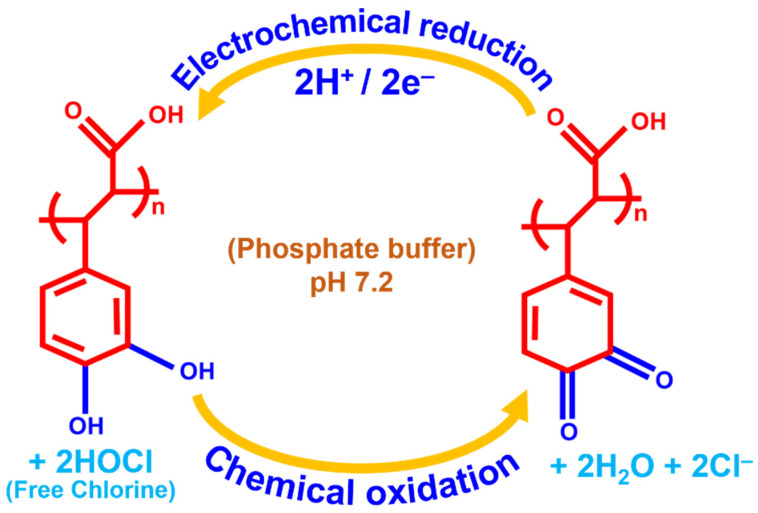
p-CFA@ERGO/GC is a possible electrocatalytic free chlorine sensing mechanism.

Caffeic acid (CFA, Scheme 1), or 3,4-dihydroxycinnamic acid, is a familiar and naturally occurring phenolic derivative compound. CFA is a solid yellow substance containing acrylic and phenolic functional moiety. CFA is a useful synthon for biosynthesis, such as lignin, a major constituent of plants and biomass of its residues [23]. CFA contains the reduced and oxidized forms of the o-hydroquinone/*o*-quinone redox couple used effectively as electrode materials for electrocatalytic applications [24]. The poly-caffeic acid (p-CFA)-coated GC electrode offered an excellent stable redox peak current (Scheme 2). The redox couple revealed the two-electron two-proton p-CFA reaction process [24]. Recently, CFA-coated electrodes have been used for the determination of various analytes such as acetaminophen [25], ascorbic acid [26], glutathione [27], uric acid [28], dopamine [29], epinephrine [30], and nicotinamide adenine dinucleotide [24]. The benefit of p-CFA as an electrode material is its *o*-quinone redox probe that facilitates the electron transfer reaction between the target analyte and electrode. The p-CFA-coated electrode revealed the commensurate electrocatalytic current and peak potential; however, it limits its electron transfer rate and the sensitivity of the analyte [25]. The peak current of electrochemically reduced graphene oxide is incorporated into the GC surface before polymer deposition on a bare substrate to better understand the p-CFA electrode reaction.

Graphene has attracted considerable attention over the last decades because of its physicochemical properties, such as electronic conductivity, surface area, thermal/chemical stability, mechanical strength, and room temperature electron mobility [31,32]. The aforementioned properties of graphene are promising for versatile applications, such as batteries [33], refractive index sensors [34], sensors [35,36,37,38,39], solar cells [40], supercapacitors [41,42], and fuel cells [43]. The graphene oxide (GO) electrochemical reduction method is a fascinating approach because of the fast and environmentally friendly way to reduce GO synthesis, which is beneficial for electrochemical sensor applications [35,36,37]. Combining the polymer–graphene composites offers an excellent polymer redox couple with carbon material with good electronic conductivity suitable for a target biomolecule selective sensor using an electrochemical method [19,44]. Electrochemically reduced graphene oxide (ERGO) provides an excellent microenvironment for the electrochemical polymerization of monomers. In addition, polymer growth on the ERGO surface prevents agglomeration [19,44]. The present work aimed to assemble p-CFA/ERGO-coated GC electrode preparation. The obtained p-CFA@ERGO/GC composite electrode was applied to the innovative electrocatalytic determination of free chlorine in drinking and tap water samples with an extensive concentration range.

## 2. Materials and Methods

### 2.1. Materials

Sodium hypochlorite, graphite, mono, and di-sodium hydrogen phosphate (NaH_2_PO_4_ and Na_2_HPO_4_) were received from Alfa Aesar, Haverhill, MA, USA. The phosphate buffer was 0.2 M (PB, pH 7.2) and was prepared by mixing NaH_2_PO_4_ and Na_2_HPO_4_. All aqueous solutions were prepared using deionized (DI) water. The HOCl/OCl stock solution was prepared using 14% (*v*/*v*) commercial hypochlorite. Before beginning the experiments, a new, free chlorine stock solution was prepared every day. The concentrations were confirmed spectrophotometrically with a wavelength (λ_max_) of 289 nm. Caffeic acid was acquired from Aldrich. The GC (1 mm thick) plates were received from Alfa Aesar to ensure p-CFA and ERGO synthesis using Raman spectroscopy, field emission scanning electron microscopy (FESEM), and X-ray photoelectron spectroscopy (XPS). 

### 2.2. Characterization

The p-CFA and ERGO surface morphology were characterized by field emission scanning electron microscopy (FESEM) (Hitachi S4800, Tokyo, Japan). Raman spectroscopy (Horiba France SAS XploRA PLUS, Lille, France) was performed with excitation at 532 nm. The surface functional moiety was verified by X-ray photoelectron spectroscopy (XPS) (Thermo Scientific K-Alpha, Boston, MA, USA). Electrochemical analyses were conducted on a galvanostat/potentiostat (Metrohm Autolab PGSTAT302N, Utrecht, The Netherlands). A conventional cell setup (three-electrode) was used for all the electrochemical experiments. The GC, saturated calomel (SCE) electrode, and platinum wire were used for the working, reference, and counter electrodes. The electrochemical experiments were conducted in an N_2_ atmosphere at room temperature (RT).

### 2.3. Fabrication of p-CFA@ERGO/GC

GO was synthesized using the Hummers’ approach [35,45]. Scheme 1 represents a schematic drawing of the p-CFA polymerization on the ERGO-coated GC electrode. First, the GC surface was polished with an emery sheet and alumina slurries in sizes of 1.0, 0.3, and 0.05 μm in range and washed. A 1 mg GO per mL sample of the ethanol solution dispersed using 5 μL (5%) Nafion^®^ as a binder was mixed and sonicated for 20 min to produce the suspension [46]. Subsequently, the GO suspension was spread over the GC surface and allowed to dry at RT. The gained GO/GC was reduced electrochemically within the voltage range of 0.0 to −1.5 V with a sweep rate of 50 mV s^−1^. The obtained electrode is indicated as the ERGO/GC. The CFA electropolymerization was carried out on the ERGO/GC surface, with the sweeps ranging from +0.10 to +0.90 V at a sweep rate of 20 mV s^−1^ in 2 mM monomer concentration in pH 4.0 (0.2 M PBS) solution [27]. The ERGO and p-CFA coatings were confirmed; similar procedures were conducted on the GC plate surface for the Raman, XPS, and FESEM studies of different modified electrodes, such as GO/GC, ERGO/GC, and p-CFA@ERGO/GC.

## 3. Results and Discussion

### 3.1. GO Electrochemical Reduction and p-CFA Formation Mechanism on the ERGO Surface

The GO/GC electrode underwent cyclic voltammetry (CV) cycles of 15 repeats to eradicate the functional oxygen moieties on the GO. The first CV trace peaked at −1.3 V, indicating a reduction in the oxygen functional groups contained in the GO [35,36]. In the second CV curve, a trace of the decreasing peak current revealed the removal of oxygen functional groups. The third scan diminished the CV trace, indicating that the GO was almost reduced to ERGO. Here, the aromatic skeleton of graphene was restored on the GC because of the removal of oxygen functional groups by the electro-reduction of the GO. 

**Figure 1:**
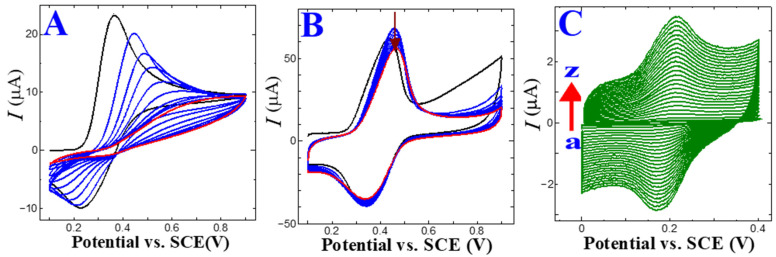
CV trace of electrochemical CFA (2 mM) polymerization at a scan rate of 20 mV s^−1^ and pH 4.0 (0.2 M PBS) on (**A**) bare glassy carbon and (**B**) ERGO/GC (black line: first cycle, blue lines: second to ninth cycles, red line: 10th cycle). (**C**) CV trace of a p-CFA@ERGO/GC-coated electrode at various scan rates from 5 to 600 mV s^−1^.

Furthermore, a potentiodynamic CFA electropolymerization was performed in pH 4 (PBS 0.2 M) supporting electrolyte medium on the bare GC and ERGO/GC electrode. The bare GC surface CFA electropolymerization CV trace revealed a lower current response than the ERGO/GC, and peak potential shifted towards the positive direction. The p-CFA/GC showed an unstable anodic and cathodic peak potential (Figure 1A). The p-CFA electropolymerization on ERGO/GC shows a high peak current and well-defined redox couple (Figure 1B) [27]. These results were attributed to the GO electro-reduction process. Some unreduced hydroxyl groups might have interacted with the carboxylic/carbonyl groups of CFA. In addition, the p-CFA@ERGO composite displayed a higher peak current than the p-CFA/GC coated electrode, indicating the excellent conductivity of the ERGO substrates (Figure 1B). 

### 3.2. p-CFA@ERGO/GC Electrochemical Study

Figure 1C shows the CV traces acquired in the p-CFA@ERGO/GC-coated electrode at pH 7.2 (PB 0.2 M). The corresponding p-CFA redox peaks yielded an anodic peak (Epa) at +0.2 V and a cathodic peak (*E_pc_*) of +0.14 V, respectively. The *τ* peak was attributed to the p-CFA catechol group oxidization as an o-quinone form, which reduced the *E_pc_* reduction process to the initial status (catechol group) [27]. The p-CFA coated ERGO/GC surface coverage (*τ*) was calculated to estimate the effective charge (*Q*) present in polymer film development according to the below Equation (4):

(4) $$\tau =\frac{Q}{nFA}$$

where *Q*, *n*, *A*, and *F* are the charges of the CV trace area (integrated), the number of electrons contributing to the p-CFA redox reaction, the GC area (geometric, 0.0707 cm^2^), and Faraday constant 96,485 C mol^−1^, respectively. The estimated τ for the p-CFA@ERGO/GC-coated electrode was 4.75 × 10^−11^ mol cm^−2^. The calculated τ for the p-CFA/ERGO-coated electrode indicates the construction of a thin polymer layer. The p-CFA@ERGO/GC electrode kinetic process was identified using different sweep rate studies in pH 7.2 (0.2 M PBS). The sweep rate was increased from 5 to 600 mV s^−1^, and the oxidation/reduction peak current gradually increased, showing that the p-CFA affords an excellent redox couple (Figure 1C). The peak current versus scan rate plot of the p-CFA@ERGO/GC-coated electrode revealed a linear increase in current with an increasing sweep, which ensured that the electrode underwent a surface-confined reaction (*R*^2^ = 0.999) (Figure S1). 

### 3.3. Electron Microscopic Study of the Modified Electrode

**Figure 2:**
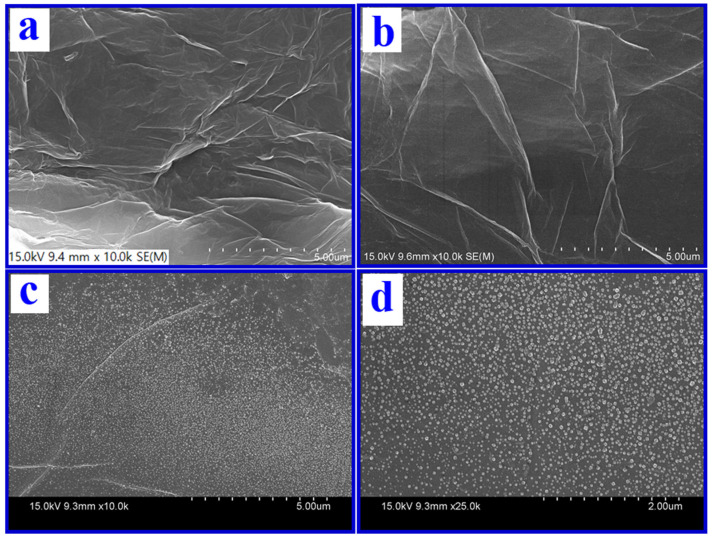
FESEM images of (**a**) GO/GC, (**b**) ERGO/GC, and (**c**,**d**) p-CFA@ERGO/GC low and high magnifications.

Figure 2a–d shows the FESEM of GO/GC, ERGO/GC, and p-CFA@ERGO/GC-plated surface coating and morphology ensured with sequential modifications. The GO-coated GC surface revealed a wavy/crumpled sheet-like structure, whereas reduced GO (ERGO) showed an intense wrinkled sheet-like morphology (Figure 2a,b) [34,35,36,45]. This behavior was attributed to the surface tension, edge instability, and two-dimensional sheet strain upon GO preparation and conversion to ERGO. In addition, the p-CFA@ERGO/GC-coated plate exhibited the cluster-like surface microstructure of polymer grown on the ERGO (Figure 2c,d). The basal and edge plane defects favor CFA monomer interaction and polymer deposition on the carbon surface because ERGO contains some unreduced oxygen groups [19]. Figure 2d shows FESEM images of p-CFA@ERGO with high magnification, confirming that p-CFA had an even distribution of polymer particles over the ERGO surface.

### 3.4. X-ray Photoelectron and Raman Spectra Studies

**Figure 3:**
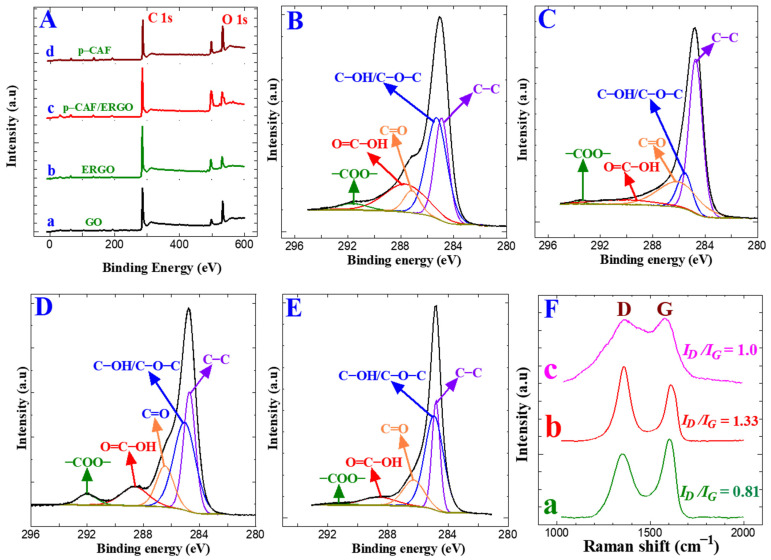
(**A**) XPS survey (a) GO/GC, (b) ERGO/GC, (c) p-CFA@ERGO/GC, and (d) p-CFA/GC, (**B**–**E**) C 1s region envelopes of (**B**) GO/GC, (**C**) ERGO/GC, (**D**) p-CFA@ERGO/GC, and (**E**) p-CFA/GC, (**F**) Raman spectra of (a) GO/GC, (b) ERGO/GC, (c) p-CFA@ERGO/GC.

XPS is generally used to find the oxidation state of elements and characterize surface changes. The electro-reduction of GO was ensured using an XPS study, and Figure 3A (a and b) shows their GO and ERGO survey spectra on GC plates. Figure 3A (a–d) presents the survey spectra of different coatings on the GC plate electrodes: GO, ERGO, p-CFA@ERGO, and p-CFA. In the survey, the trace showed the two peaks of C 1 s (sp^2^ carbon) and O 1 s (–O–, –OH, COOH functional groups) at 285 and 531 eV, respectively [34,45]. The carbon/oxygen ratio is vital to validating the graphite oxidation through an exfoliation reaction. The carbon/oxygen peak intensity ratio for GO and ERGO was 1.4 and 2.11. The high C/O ratio value of the ERGO highlights the removal of the functional oxygen moiety upon the electro-reduction of GO [45]. This study confirmed the successful electro-reduction of GO. Figure 3B–E shows the deconvoluted high-resolution XPS spectra of GO, ERGO, p-CFA@ERGO, and the p-CFA-coated GC plate. The C 1 s region, deconvoluted into five envelopes at 284.6, 285.8, 286.0, 288.5, and 291.6 eV, was assigned to C–C, C–O–C/C–OH, C=O, COOH, and COO–, respectively. The ERGO’s C(OH) and other oxygen groups showed low areas under the GO envelopes, which confirmed the successful electro-reduction of the as-prepared GO (Figure 3B,C). On the other hand, the C–OH area under the envelope of the p-CFA@ERGO composite electrode revealed a 20% higher value. Hence, the p-CFA contains catechol (–OH, COOH) groups and an effective polymer film on the ERGO. The p-CFA coating on the bare GC electrode surface was tested with a controlled experiment. The results are displayed in Figure 3E. p-CFA/GC shows that the region envelopes’ binding energies at 284.6, 285.0, 286.3, 288.6, and 291.4 eV correspond to the –C–C–, C–O–C/C–OH, C=O, –COOH, and –COO–. These binding energies resemble the positions with the p-CFA@ERGO region, which ensures the p-CFA ability to form on carbon and ERGO substrates. Table S1 lists the detailed functional groups of each sample, binding energy, and area of the deconvoluted envelopes. 

The electro-reduction of GO to ERGO was confirmed by Raman spectra, widely used to evaluate carbon materials’ order and disorder levels. Figure 3F (a–c) displayed the Raman spectra of the GO/GC, ERGO/GC, and p-CFA@ERGO/GC plate-coated materials. Carbon-based materials generally reveal two significant D and G peaks. The D and G peaks were attributed to the A_1g_ symmetry breathing mode k-point (phonons) and E_2g_ (phonon) mode sp^2^ carbons, respectively. The GO-coated electrode revealed two bands at 1594 cm^−1^ and 1351 cm^−1^, ascribed to the G (graphitic) and D (defect) peaks, respectively (Figure 3F (a)). Similarly, the ERGO-coated electrode also showed the G and D bands at 1587 and 1344 cm^−1^, respectively (Figure 3F (b)). The D band intensity increased compared to the GO. This result also guaranteed the GO presence on the bare GC surface and electro-reduction reaction. The G band peaks shift to a low wavenumber, indicating a basal plane defect, increasing and restoring the aromatic graphene skeleton structure [35,36,47]. The polymer-coated p-CFA@ERGO/GC electrode revealed G and D bands at 1564 and 1344 cm^−1^. The *I_D_*/*I_G_* ratio provides information about the surface modification of carbon-based materials, such as chemical changes or disorders [47]. The measured *I_D_*/*I_G_* values were 0.81, 1.33, and 1.0, corresponding to the GO/GC, ERGO/GC, and p-CFA@ERGO/GC electrodes, respectively. The high *I_D_/I_G_* ratio of the ERGO indicates the aromatic skeleton structure regeneration as an sp^2^ graphene backbone. Moreover, the p-CFA@ERGO peak intensity *I_D_*/*I_G_* ratio decreased to 1.0, which is lower than the ERGO/GC electrode, indicating the recovery of the polymer catechol aromatic unit on ERGO [48]. Table S2 lists the GO, ERGO, and p-CFA@ERGOs’ G and D peak assignments and the *I_D_*/*I_G_* values. The average crystalline (*La*) size of the carbon-based materials was estimated by the *I_D_*/*I_G_* ratio value using Equation (5) [38]:

(5) $$L_{a}\left(\mathrm{nm}\right)=2.4\times 10^{-10}\;\lambda_{l}^{4}{\left(\frac{I_{D}}{I_{G}}\right)}^{-1}$$

where *λ* and *La* are the laser wavelength (nm) and the average crystalline size, respectively. The calculated values of *La* for the GO/GC, ERGO/GC, and p-CFA@ERGO/GC-coated electrodes were 22.8, 13.9, and 19.2 nm, respectively. The decreasing average crystallite size from GO to ERGO indicates a decrease in sp domain size due to the electro-reduction in GO. After the p-CFA coating on ERGO, the *La* value increased owing to the p-CFA aromatic moiety.

### 3.5. Electrochemical Impedance Spectroscopy (EIS)

**Figure 4:**
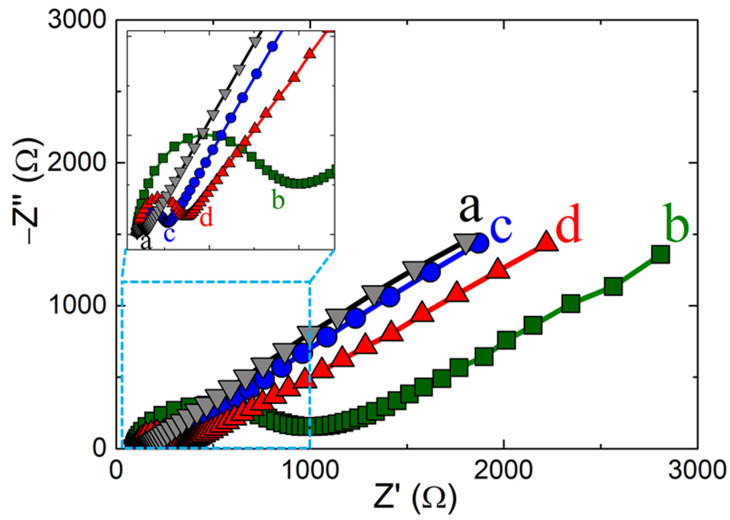
Electrochemical impedance spectroscopy of (a) bare GC, (b) p-CFA/GC, (c) ERGO/GC, and (d) p-CFA@ERGO/GC-modified electrodes in 0.1 M KCl containing 5 mM [Fe(CN)_6_]^4−/3−^.

Electrochemical impedance spectroscopy (EIS) is a sensitive technique for examining the changes in the electrode probe. Figure 4 shows the EIS results for different electrodes in the 5 mM ferro-/ferricyanide ([Fe(CN)_6_]^4−/3−^)-containing 0.1 M potassium chloride-supporting electrolyte within a range frequency of 0.01 to 100 kHz. The *x*-axis intercept point of the Nyquist plots at the high-frequency section relates to the electron transfer-controlled process. In the medium frequency semicircle, the diameter of the circle portion was attributed to the charge-transfer (*R_ct_*) resistance, representing the surface-changed electrode electron transfer rate. The linear portion at low frequency was attributed to the diffusion-controlled rate determination [25,49]. The estimated *R_ct_* values were 148, 800, 164, and 350 Ω, representing the bare GC, p-CFA/GC, ERGO/GC, and p-CFA@ERGO/GC-coated electrodes (Figure 4). The charge transfer values of p-CFA/GC and p-CFA@ERGO/GC were higher than the bare GC electrode. This result was attributed to the p-CFA negative charge hydroxyl and carboxylic group with ferro/ferri redox probe repulsion. Similarly, ERGO/GC showed a higher *R_ct_* than the bare GC, representing the repulsive nature of the unreduced −OH group with the ferro/ferri-probe. The ERGO/GC shows a lower *R_ct_* than the p-CFA and p-CFA@ERGO electrodes, highlighting the ERGO’s conductive nature. In contrast, p-CFA@ERGO/GC showed a comparably high *R_ct_*, confirming an increase in the repulsive nature after the p-CFA modification. This is because the hydroxyl groups of p-CFAs and unreduced hydroxyl groups of the ERGO are combined with the repulsive impact with the ferro/ferri-probe. Therefore, the electrocatalytic method using the p-CFA@ERGO/GC probe may be suitable for free chlorine determination. 

### 3.6. Electrocatalytic Free Chlorine Determination

**Figure 5:**
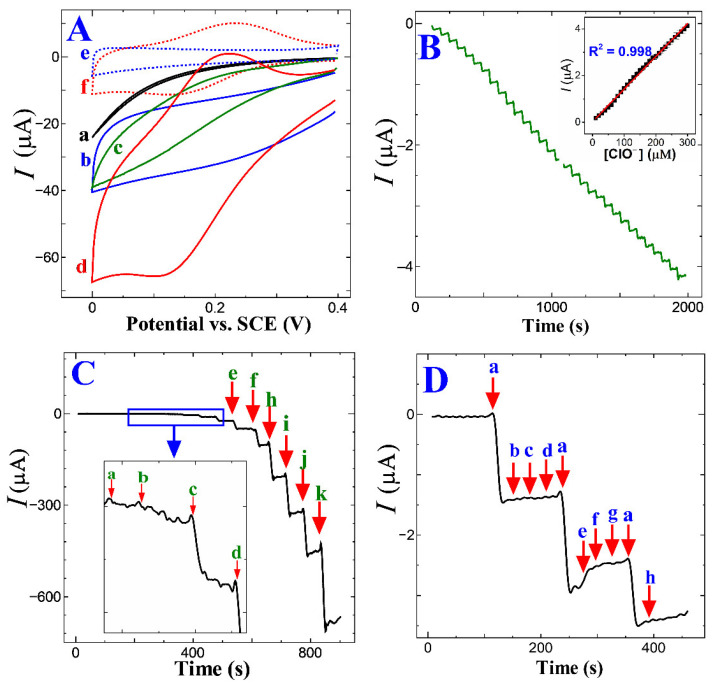
(**A**) CV trace on (a) bare GC, (b) ERGO, (c) p-CFA, and (d) p-CFA@ERGO/GC in 5 mM free chlorine and absence of analytes (e) ERGO/GC and (f) p-CFA@ERGO/GC at a scan rate of 50 mV s^−1^ in PBS 7.2 (0.2 M); (**B**) Chronoamperometric i–t trace for every minute successive 10 μM free chlorine addition on the p-CFA@ERGO/GC and applied operating voltage at +0.17 V (inset: corresponding current vs. free chlorine concentration); (**C**) wide-range chronoamperometry free chlorine measurement from (a) 20, (b) 50, (c) 100, (d) 200, (e) 500, (f) 1000, (g) 2000, (h) 5000, (i) 7000, (j) 10000, and (k) 20000 μM, and (**D**) p-CFA@ERGO/GC selectivity test with 10 µM free chlorine in (a) pure water and in the presence of 1 mM of other interfering ions such as (b) SO_4_^2−^, (c) CO_3_^2−^, (d) Cl^−^, (e) SO_3_^2−^, (f) urea, (g) ethanol, and (h) NO_3_^−^.

Figure 5A shows the CV trace response of the GC, ERGO/GC, p-CFA/GC, and p-CFA@ERGO/GC-coated electrode in a pH 7.2 (PBS 0.2 M) medium absence and the presence of free chlorine. The bare GC and ERGO showed no significant free chlorine (5 mM) reduction peaks; however, only an increase in the background current was observed (Figure 5A (a and b)). The p-CFA/GC and p-CFA@ERGO/GC probes revealed an increasing free chlorine reduction current, indicating that an electrocatalytic reduction occurred. The p-CFA@ERGO/GC electrode-free chlorine reduction peak current response was 12.4-, 2.0-, and 2.7 times higher than that of the bare GC, ERGO/GC, and p-CFA/GC, respectively. Compared to the CV trace of p-CFA/GCs, p-CFA@ERGO/GC observed well-defined anodic and cathodic peaks at potentials of +0.21 and +0.17 V, respectively. Because p-CFA catechol oxidized the two-electron and two-proton product of *o*-quinone with this process, the quinoid structure offered a delocalized electron that effectively transferred with the ERGO conductive substrate, unlike the bare GC-coated p-CFA electrode. The p-CFA@ERGO/GC electrocatalytic sensor is explained as follows: first, catechol moiety hydroxyl groups are chemically oxidized by free chlorine, producing the *o*-quinone, water, and chloride ions. 

In the next step, *o*-quinone is reduced electrochemically using a 2e^−^/2H^+^ addition that repeatedly regenerates the catechol hydroxyl moiety. Scheme 2 represents a better understanding of the electrocatalytic mechanism. As produced, the *o*-quinone electrochemical reduction reaction current response was monitored. The developed sensor mechanism supported earlier literature reports of hypochlorous acid/OCl^−^ reduced by catechol and chemically converted to *o*-quinone [50]. Kumar et al. electrochemically synthesized a polydopamine/graphene electrode using a similar dopamine hydroxyl group for *o*-quinone product conversion, which also supports the present electrode mechanism [19]. The p-CFA/ERGO composite electrode provided a high current that reduced graphene oxide, making it a facile microenvironment for the redox polymer with target-free chlorine (Figure 5A (d and f)).

### 3.7. Chronoamperometric Detection of Free Chlorine

Figure 5B shows the optimized experimental conditions p-CFA@ERGO/GC caused to chronoamperometry (i-t) curve performance in a pH 7.2 (0.2 M PBS) medium with a free chlorine operating reduction potential of +0.17 V. The free chlorine reduction peak current increased steadily on each successful 10 µM injection. Free chlorine additions at every one-minute interval increased the current, which was stabilized within three seconds. The linear increment of the free chlorine concentration from 10.0 µM to 300.0 µM increased the current versus concentration plot (Figure 5B inset) and found a correlation coefficient of 0.998. The proposed p-CFA@ERGO/GC electrode widespread free chlorine concentration detection ability was tested from the 20 µM to 20 mM range and obtained an excellent linearity coefficient of 0.9895 (Figure 5C and Figure S2). The slope showed a superior sensitivity of 0.0361 µA µM^−1^. The lowest detection limit (LOD) was estimated using the following equation: LOD = 3*σ*/*m*, because *σ* is the standard deviation of the current response at a blank PBS solution, and *m* refers to the slope of the calibration trace. The calculated LOD of free chlorine for p-CFA@ERGO/GC was 0.03 µM. As developed, sensor electrode performance, such as the pH of the supporting electrolyte, operating potential, linear range, sensitivity, and LOD, were compared in the previous reports (Table 1). In earlier studies, the innovative p-CFA@ERGO composite electrode revealed a comprehensive concentration-free chlorine sensing capability and low LOD [15,16,17,18,19,20]. The compatible interfacial interaction between polymer and graphene confers superior electrochemical performance. ERGO contains some unreduced oxygen functional groups that interact with the caffeic acid −OH by intermolecular interaction, which reinforces the p-CFA uptake on the electrode surface. Thus, the p-CFA@ERGO composite electrode emphasized the high surface coverage that offered wide-range free chlorine detection, comparable sensitivity, low LOD, and storage stability.

### 3.8. Selectivity, Storage Stability, and Reproducibility Test

The p-CFA@ERGO composite electrode selectivity/tolerance test was conducted with typical water samples containing ions such as sulfite (SO_4_^2−^), carbonate (CO_3_^2−^), sodium chloride (NaCl), and sulfite (SO_3_^2−^), urea, ethanol, and nitrate (NO_3_^−^) (Figure 5D). The i-t current tolerability of the p-CFA@ERGO electrode with 10 µM free chlorine was measured by adding each 1 mM of all interfering ions, obtaining no significant current alteration of the target analyte. This observation showed that the proposed electrode has excellent interfering ion tolerability. Here, only sulfite ions showed a slight change in current in the presence of free chlorine. Long-term storage capability was tested for p-CFA@ERGO/GC after thirty days of a five-set of chronoamperometry with the 10 µM analyte. The measured relative standard deviation (RSD) was 5.5% (*n* = 5). The free-chlorine detection repeatability was tested with five p-CFA@ERGO/GC electrodes, getting 4.2% RSD. The five p-CFA@ERGO/GC set electrode fabricated and examined the free chlorine detection with the same concentration obtained RSD was 4.5%, indicating the outstanding reproducibility of the proposed electrode.

### 3.9. Realistic Drinking and Tap Water Sensors

The real-time applicability of the as-fabricated p-CFA@ERGO/GC electrode was substantiated by measuring the free chlorine concentration in water samples. The model sample, such as tap water and commercial drinking water, was collected, and the pH of the samples was adjusted to 7.2 using a 0.2 M PBS solution. The commercial drinking and tap water samples containing free chlorine levels were tested by the standard addition method. Table 2 lists the obtained recovery values. The recovery values of free chlorine in commercial (drinking) and tap waters were 98.5 and 99.9%. The obtained recovery values were satisfactory in realistic water sample analysis. The present sensor electrode performance was validated using the well-known *N,N*′-diethyl-*p*-phenylenediamine (DPD) reagent colorimetric technique. The present sensor electrode revealed that free chlorine measurements in commercial (drinking) and tap waters were 9.9, 19.9, 9.85, and 19.95 µM. These values agree with the regular DPD colorimetric results of 10.1, 19.8, 9.9, and 20.40 µM. This study proved that the assembled p-CFA@ERGO/GC composite electrode might be suitable for real-time free chlorine detection in water samples.

## 4. Conclusions

The potentiodynamic method was used for poly(caffeic acid) deposition on the electro-reduced graphene oxide/glassy carbon electrode surface. FESEM, Raman, XPS, and EIS confirmed the successful fabrication of polymer/ERGO. The poly(caffeic acid)/ERGO composite electrode containing a catechol/*o*-quinone redox probe was applied to the electrocatalytic water disinfectant agents, such as free chlorine sensors, as the obtained p-CFA@ERGO/GC sensor electrode revealed an outstanding free chlorine detection range (20 µM to 20 mM), sensitivity (0.0361 µA µM^−1^), and the lowest LOD value (0.03 µM). Finally, p-CFA@ERGO/GC was applied successfully to measure the commercial and tap water samples and found good recoveries from 98.5% to 99.9%. These results are satisfactory with the DPD method, and the results agree that the proposed p-CFA@ERGO/GC might be suitable for future free chlorine sensor probes.

## Figures and Tables

**Table 1:** p-CFA@ERGO/GC free chlorine sensing performance compared with previous reports.

Electrode	pH	Applied Potential (V)	Type of Reaction	Linear Range (μM)	Sensitivity (μA μM^−1^)	Detection Limit (μM)	Ref.
Graphite electrode	10	+1.03	Oxidation	19.4–5830	−	19.4	[16]
BDD electrode ^a^	8	+1.40	Oxidation	0.39–1940	0.038	0.16	[15]
Polymelamine/SPCE ^b^	7	+0.125	EC	10–7000	0.058	5.5	[18]
Au microelectrode	5.5	+0.35	Reduction	3.89–97.17	0.012	0.39	[17]
Polydopamine/ERGO/GC	7.2	+0.15	EC	9.9–215.2	0.0071	0.044	[19]
polyMnTAPP-Au/GC ^c^	10.4	−0.15	Reduction	24–10,700	−	24.0	[20]
**p-CFA@ERGO/GC**	**7.2**	**+0.17**	**EC’**	**20–20,000**	**0.0361**	**0.030**	**This work**

^a^ Boron-doped diamond electrode; ^b^ Screen printed electrode; ^c^ Poly manganese tetra (o-amino phenyl) porphyrin; EC’: Electrocatalytic reaction.

**Table 2:** Determination of free chlorine in drinking and tap water samples using the p-CFA@ERGO/GC-modified electrode (the values were obtained from five samples (*n* = 5) for each water).

Samples	Free Chlorine Concentration before Addition (µmol L^−1^)	Electrochemical Method			DPD Method		
		Added (µmol L^−1^)	Found (µmol L^−1^)	Recovery (RSD) (%)	Added (µmol L^−1^)	Found (µmol L^−1^)	Recovery (RSD) (%)
Drinking water-1	0	10	9.9	99.0 (1.428)	10	10.1	101 (2.562)
Drinking water-2	0	20	19.9	99.5 (0.683)	20	19.8	99.0 (1.241)
Tap water-1	0.50	10	9.85	98.5 (0.117)	10	9.9	99.0 (0.354)
Tap water-2	0.56	20	19.95	99.9 (2.177)	20	20.40	102 (1.935)

## Data Availability

The data presented in this study are available in the lab research notebook in Yeungnam University.

## Supplementary Materials

The following are available online at https://www.mdpi.com/article/10.3390/nano13010151/s1. Figure S1. Plot of the anodic peak current vs. scan rate; Figure S2. Amperometric i-t calibration plot; Table S1. Measured binding energies and areas for standard chemical states of carbon C 1 s region of XPS; Table S2. *I_D_/I_G_* and C/O ratios obtained from the Raman and XPS spectra.
