# Supplementary material for: Poly(caffeic acid) Redox Couple Decorated on Electrochemically Reduced Graphene Oxide for Electrocatalytic Sensing Free Chlorine in Drinking Water
Source: Nanomaterials (Basel). 2022 Dec 28;13(1):151. doi: 10.3390/nano13010151 (PMC9824798; doi:10.3390/nano13010151)
Supplement: Supplementary file 1 [file nanomaterials-13-00151-s001.zip › nanomaterials-1981080-supplementary.pdf]

Supporting Information

Poly(caffeic acid) redox couple decorated on electrochemically reduced graphene oxide for electrocatalytic sensing free chlorine in drinking water

Srinivasan Kesavan†, Deivasigamani Ranjith Kumar†, Ganesh Dhakal, Woo Kyoung Kim, Yong Rok Lee, Jae-Jin Shim*

School of Chemical Engineering, Yeungnam University, 280 Daehak-ro, Gyeongsan, Gyeongbuk 38541, Republic of Korea

---

†These authors contributed equally to this work.

*Correspondence *E-mail address*: jjshim@yu.ac.kr (J.-J. Shim).

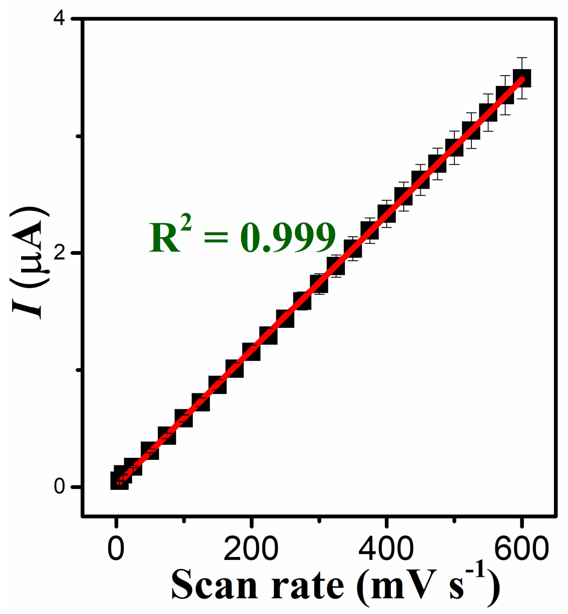

Figure S1. A plot of the anodic peak current vs. scan rate.

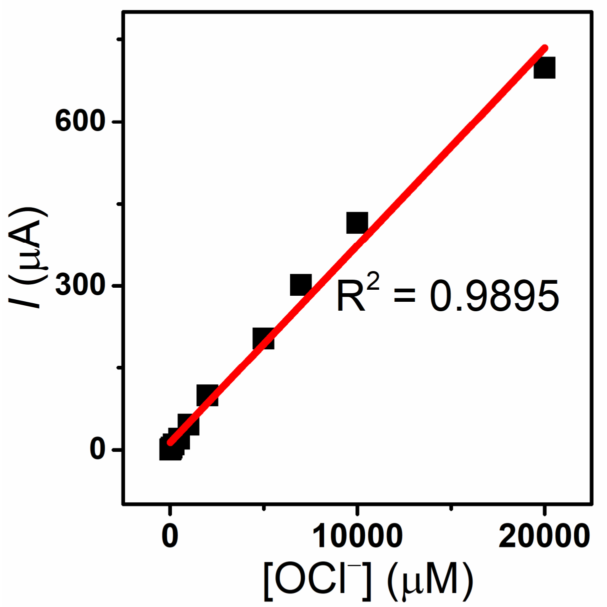

Figure S2. Amperometric i–t curve for the determination of free-Cl on the p-CFA@ERGO/GC modified electrode at a wide concentration range: (a)

0.02, (b) 0.05, (c) 0.1, (d) 0.2, (e) 0.5, (f) 1, (g) 2, (h) 5, (i) 7, (j) 10, and (k) 20 mM addition.

Table S1. Measured binding energies and areas for common chemical states of carbon in the C 1s region of XPS.

Sample	Chemical state	Binding energy (eV)	Area
	C–C	284.6	63,572
	C–OH/C–O–C	285.3	92,322
GO/GC	C=O	287.1	15,562
	O=C–OH	288.0	51,909
	–COO–	291.6	8,267
	C–C	284.6	131,220
	C–OH/C–O–C	285.5	32,805
ERGO/GC	C=O	286.0	66,100
	O=C–OH	288.8	18,978
	–COO–	292.8	1,646
	C–C	284.6	34,555
	C–OH/C–O–C	285.0	69,707
p-CAF/GC	C=O	286.3	22,098
	O=C–OH	288.6	10784
	–COO–	291.4	1,336
	C–C	284.6	29,116
	C–OH/C–O–C	285.2	39,119
p-CAF@ERGO/GC	C=O	286.5	14,558
	O=C–OH	288.5	10,560
	–COO$^-$	292.0	4,317

Table S2. I_D/I_G and C/O ratios obtained from the Raman and XPS spectra.

Sample	Raman spectral data			XPS
	D band (cm^{-1})	G band (cm^{-1})	I_D/I_G	C/O ratio
GO/GC	1351	1594	0.81	1.40
ERGO/GC	1344	1587	1.33	2.11
p-CAF@ERGO/GC	1344	1564	1.0	0.47
